# Effect of epigenetics on vitamin D levels: a systematic review until December 2020

**DOI:** 10.1186/s13690-023-01122-2

**Published:** 2023-06-15

**Authors:** Ali Forouhari, Motahar Heidari-Beni, Shaahin Veisi, Parnian Poursafa, Roya Kelishadi

**Affiliations:** 1 Isfahan Eye Research Center, Department of Ophthalmology, Isfahan University of Medical Sciences, Isfahan, Iran; 2 Department of Nutrition, Child Growth and Development Research Center, Research Institute for Primordial Prevention of Non-Communicable Disease, Isfahan University of Medical Sciences, Isfahan, Iran; 3 School of medicine, Isfahan University of Medical Sciences, Isfahan, Iran; 4 Interdisciplinary Neuroscience, Interdisciplinary Center for Neuroscience, Goethe University, Frankfurt, Germany; 5 Department of Pediatrics, Child Growth and Development Research Center, Research Institute for Primordial Prevention of Non-Communicable Disease, Isfahan University of Medical Sciences, Isfahan, Iran

**Keywords:** DNA methylation, Epigenetics, Vitamin D, 25-Hydroxyvitamin D3

## Abstract

**Background:**

The association between epigenetic modification of the genes involved in the vitamin D metabolic pathway and vitamin D metabolites’ status has been elucidated incompletely. This study aims to review the studies on the mentioned association and create a brighter view of this topic.

**Methods:**

A systematic literature search was conducted in Medline database (PubMed), Scopus, and Web of Science up to the end of November 2020. Original articles which reported the effect of epigenetic alteration—methylation level or its changes—of genes involved in vitamin D regulation on the vitamin D metabolites serum level or its changes were included. The National Institutes of Health (NIH) checklist was used to assess the quality of included articles.

**Results:**

Among 2566 records, nine reports were included in the systematic review according to the inclusion and exclusion criteria. Studies discussed the contribution of methylation status of members of the cytochrome P450 family (CYP2R1, CYP27B1, CYP24A1), and Vitamin D Receptor (VDR) genes to vitamin D level variance. CYP2R1 methylation status could regulate the contributing factors affecting the vitamin D serum level and predict response to vitamin D supplementation. Studies revealed that impaired methylation of CYP24A1 occurs in response to an increase in serum level of 25-hydroxyvitamin D (25(OH)D). It is reported that the association between methylation levels of CYP2R1, CYP24A1, and VDR genes and 25(OH)D level is not affected by the methyl-donors bioavailability.

**Conclusions:**

The epigenetic modification of the vitamin D-related genes could explain the vitamin D levels variation among populations. Large-scale clinical trials in various ethnicities are suggested to find the effect of epigenetics on vitamin D response variation.

**Registration:**

The systematic review protocol was registered on PROSPERO (registration number: CRD42022306327).

**Supplementary Information:**

The online version contains supplementary material available at 10.1186/s13690-023-01122-2.

## Background

Vitamin D is a steroid hormone with crucial roles in calcium hemostasis and different extra-skeletal pathways. In humans, vitamin D_3_ production initiates from 7-dehydrocholesterol (7-DHC) in the skin. The concentration of 7DHC depends on the activity of the 7 Dehydrocholesterol Reductase (DHCR7) enzyme, which converts 7DHC into cholesterol [1, 2]. Afterward, vitamin D requires two hydroxylation stages for turning to the active hormonal format (1,25(OH)_2_D). 25-hydroxyvitamin D (25(OH)D) is the most detectable vitamin D metabolite in humans and is used for determining the status of vitamin D in individuals. Several enzymes are known to be involved in 25-hydroxylation, including CYP2R1, CYP27A1, and CYP3A4—members of the cytochrome P450 family [3, 4]. Despite the variation of enzymes in 25-hydroxylation, CYP27B1 is recognized as the only 1α-hydroxylase in humans [5]. CYP24A1 is the catabolic enzyme that regulates the metabolic pathway and prevents toxic amounts of vitamin D metabolites [6].

More than 11,000 genes were known as the target of 1,25(OH)_2_D [7]. The complex of vitamin D receptor (VDR), 1,25(OH)_2_D, and retinoid X receptor (RXR) interact with gene response elements, and after recruitment of co-regulatory factors [8], modulation of gene expression would be achieved [9]. Enrolled co-regulatory factors and epigenetic modifiers (methyltransferases, histone acetyltransferases, and factors with histone acetylase activity) lead to varied responses of a specific gene to the 1,25(OH)_2_D in different cells and tissues.

Epigenomic studies evaluated the effects of chromatin-modifying and remodeling enzymes which act through interpretation, addition, or removal of post-translational DNA methylation or histone modification in the absence of genomic alterations [10–12]. It is shown that methylation of cytosine residues (the cytosine that is 5’ to guanine) of CpG islands (clusters of CpGs) located at the promoter region results in gene silencing. Regulation of chromatin accessibility to transcription factors via modification of histone protein tails (including methylation and acetylation) is another part of epigenetic alterations [13].

Epigenetics and vitamin D status is a developing field of research. Although the epigenetic effect of vitamin D on the transcription of target genes has been discussed, the effect of epigenetic modification on vitamin D level and its bioavailability has still been investigated incompletely [14–20]. This systematic review was conducted to summarize the literature that has assessed the association between epigenetic modification of genes involved in the vitamin D metabolic pathway and the status of vitamin D metabolites.

## Methods

### Search strategy

The current systematic review study was conducted according to the PRISMA 2020 (Preferred Reporting Items for Systematic Reviews and Meta-Analyses) statement [21]. The protocol was registered on PROSPERO (ID: CRD42022306327). A systematic literature search was conducted in the Medline database (PubMed), Scopus, and Web of Science until the end of November 2020. The following search terms were used: ((“epigenetic” OR “epigenomic” OR “epigenomics” OR “methylation” OR “DNA methylation” OR “acetylation” OR “DNA acetylation”) AND ((“vitamin D” OR “25(OH)D” OR “25-hydroxyvitamin D” OR “hydroxycholecalciferols” OR “hypovitaminosis D” OR “vitamin D deficiency” OR “1,25(OH)2D” OR “cholecalc*” OR “serum vitamin D” OR “25-hydroxyvitamin D3” OR “Calcitriol”)). The reference lists of the included studies were checked to find undetected relevant studies.

### Inclusion and exclusion criteria

Studies that assessed the effect of epigenetic modifications—methylation level or its changes—of genes involved in the vitamin D metabolic pathway on vitamin D metabolites status—serum level or its changes—and were reported in the English language were selected. Original research studies—with any design—that reported the mentioned association in humans, without any restriction of ethnicity, gender, race, and year of publication, were included. Duplicate publications and studies without enough data and information were excluded. Two expert reviewers independently assessed the title and abstract of studies to evaluate their inclusion eligibility. In case of disagreement between reviewers, the third reviewer (principal investigator) made the decision. Afterward, full-text of the articles were screened based on the inclusion and exclusion criteria, and any discrepancy between the reviewers was resolved by the principal investigator.

### Data extraction and quality assessment

Following the full-text assessment of reports, reviewers extracted the following data from the included studies; first author name, year of publication, type of study, location of the studied population and their ethnicity, any specific characteristics of the studied population, the association that is studied, total sample size, evaluated gene, CpG ID, chromosome number and the CpG position on the chromosome, location type of the CpG site, association statistics (R^2^, r, and β), false discovery rate (FDR), and p-value. A third reviewer assessed all the extracted information. Quality of the included studies was assessed by the National Institutes of Health (NIH) study quality assessment tool [22]. Two separate reviewers scored each study based on the NIH tool. In cases of disagreement, the third reviewer’s opinion was sought.

## Results

**Fig. 1:**
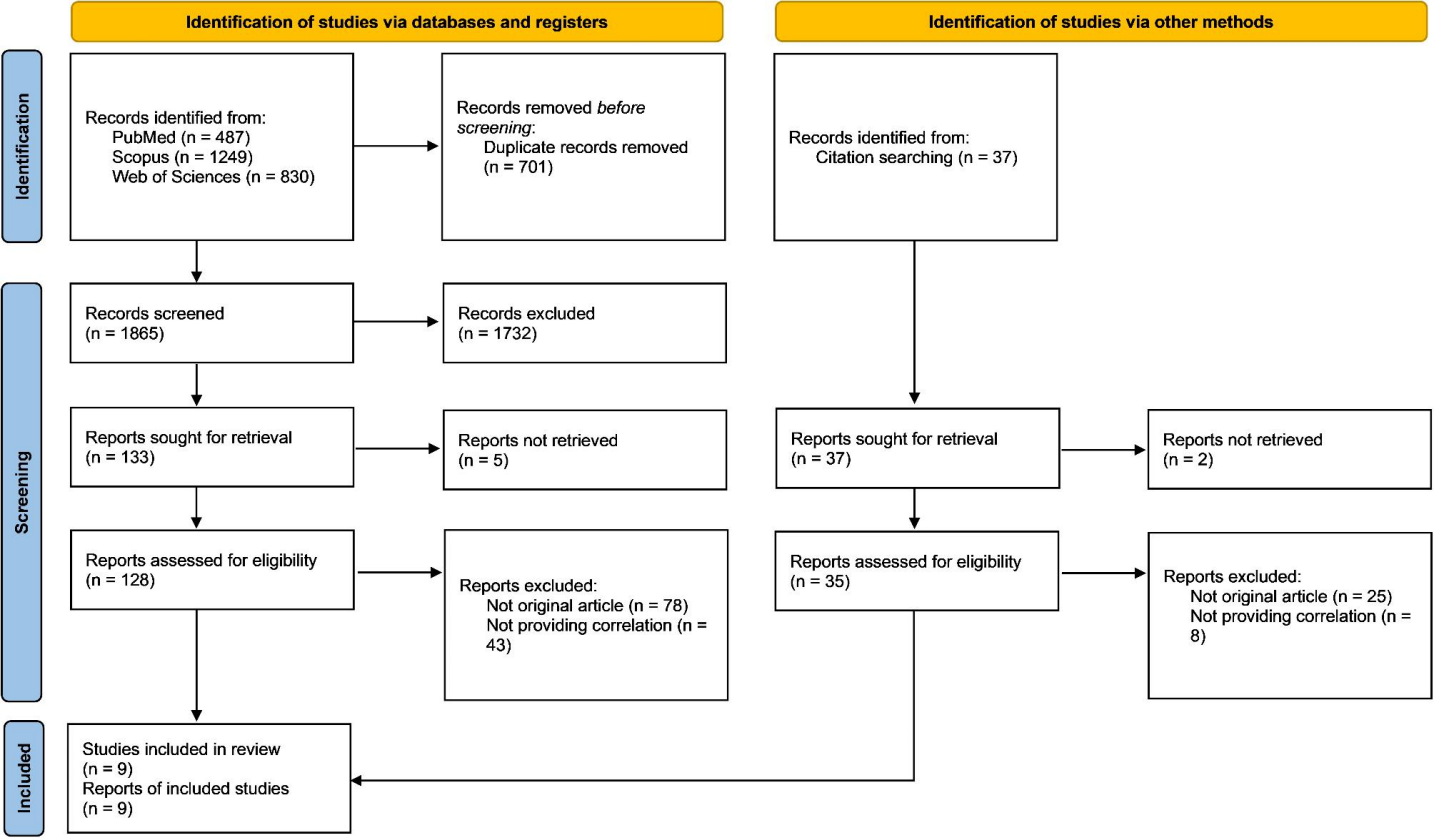
Flow diagram of the study selection process (PRISMA 2020)

The flow diagram of the study selection process is shown in Fig. 1. The initial search identified 2566 records, and 1865 of them remained after excluding duplicates. After screening the title and abstracts, 128 reports remained for further assessment. The full texts of the reports were reviewed carefully, and finally, nine research studies were included in the systematic review. Characteristics of included studies are shown in Table 1. Quality assessment of the included studies is reported in Tables 2, 3 and 4.

**Table 1:** Characteristics of the included studies according to the gene methylation sites and its effect on vitamin D levels

First author	Year of publication	Study type	Country /Ethnicity	Population-specific characteristics	Correlation between	Total number	Gene	CpG	CpG ID	Chromosome: position	Location type	Coefficient of determination (R2)	Correlation coefficient (r)	Beta coefficient (β)	FDR	*P*-value
Wjst et al., (27)	2010	Cross-sectional	Germany		25(OH)D3 level with methylation rate of CpG sites	383	CYP27B1	CpG 15–17 ^b^				0.11				All *p-*values < 0.05
O’Brien et al., (28)	2018	Case-cohort sample	USA/ Non-Hispanic white	All cases were women	25(OH)D level and with methylation level of CpGs	1270	RXRA	cg21201924		9: 137,251,825	Body			−0.02 ^a^		< 0.001
							RXRA	cg02127980		9: 137,252,116	Body			−0.015 ^a^		< 0.001
							NADSYN1	cg17559402		11: 71,187,890	Body			−0.02 ^a^		0.003
							RXRA	cg02059519		9: 137,250,935	Body			−0.01 ^a^		0.003
							GC	cg09997530		4: 72,636,217	Body			0.02 ^a^		0.005
							RXRA	cg04329455		9: 137,215,364				0.01 ^a^		0.007
							NADSYN1	cg00268518		11: 71,164,106	TSS200			0.01 ^a^		0.008
							NADSYN1	cg03146219		11: 71,189,514	Body			−0.01 ^a^		0.009
							RXRA	cg13510651		9: 137,227,772	Body			−0.01 ^a^		0.01
							DHCR7	cg03490288		11: 71,146,658	Body			0.02 ^a^		0.01
							NADSYN1	cg05785753		11: 71,189,490	Body			−0.01 ^a^		0.01
							RXRA	cg13687497		9: 137,249,839	Body			−0.01 ^a^		0.01
							NADSYN1	cg07793224		11: 71,183,180	Body			0.02 ^a^		0.02
							DHCR7	cg26044621		11: 71,145,665	3´ UTR			0.01 ^a^		0.02
							GC	cg04837494		4: 72,608,149	3´ UTR			0.01 ^a^		0.03
							RXRA	cg14236758		9: 137,252,129	Body			−0.009 ^a^		0.03
							NADSYN1	cg04774822		11: 71,165,839	Body			−0.009 ^a^		0.03
							DHCR7	cg16151558		11: 71,159,853	TSS1500			0.01 ^a^		0.04
							CYP27B1	cg20372759		12: 58,162,287	TSS1500			−0.01 ^a^		0.04
							GC	cg24806812		4: 72,635,202	Body			0.01 ^a^		0.04
							RXRA	cg14154547		9: 137,293,309	Body			−0.007 ^a^		0.04
							DHCR7	cg07099121		11: 71,146,096	3´ UTR			−0.01 ^a^		0.04
							NADSYN1	cg16910670		11: 71,215,361				−0.01 ^a^		0.05
Harvey et al., (31)	2014	Cohort	UK	Mothers and their newborns	Maternal free 25(OH)-vitamin D index (ratio of serum 25(OH)D to DBP concentrations) measured at 34 weeks gestation with cord RXRA percent methylation	64	RXRA	CpG 4/5 ^b^		9: 136355593,600 +				-3.29		0.03
Novakovic et al., (30)	2012	Cohort	Australia	Mothers and their newborns	Maternal 25(OH)D concentrations (at the birth of the child) with absolute CYP24A1 methylation	88	CYP24A1					0.01				
					Maternal 25(OH)D concentrations with CYP24A1 methylation with-in pair discordance	87	CYP24A1					0.001				
					CYP24A1 promoter methylation variation with variation in neonatal 25(OH)D concentrations	71	CYP24A1					0.02				
					Neonatal 25(OH)D concentrations with the mean concentration of CYP24A1 promoter methylation	147	CYP24A1					1E-06				
					Neonatal 25-OH‐D discordance with CYP24A1 methylation discordance in monozygotic pairs	90	CYP24A1					0.02 ^c^				0.2
						85		CpG_1 ^d^				0.02				0.14
						87		CpG_2.3.4 ^d^				0.01				0.31
						81		CpG_5 ^d^				0.02				0.23
						87		CpG_7.8 ^d^				0.01				0.46
						86		CpG_12.13 ^d^				0.0002				0.9
						89		CpG_17 ^d^				0.01				0.3
						72		CpG_18 ^d^				0.06				0.04
						81		CpG_20.21 ^d^				0.03				0.15
						87		CpG_24 ^d^				0.002				0.69
Chen et al., (24)	2020	Randomized clinical trial	USA/ African Americans	Overweight/obese and serum 25(OH)D concentrations of ≤ 50 nmol at the time of screening	Baseline CpG methylation with serum 25(OH)D response	64	OSBPL5	cg07873128		CHR: 11	Body			0.02 ^f^	0	< 0.001
					Changes in methylation level with changes in 25(OH)D response		OSBPL5	cg07873128		CHR: 11	Body					0.6
					Changes in methylation level with changes in 25(OH)D response		CYP24A1	cg06368932			1stExon, 5’UTR					0.04
					Baseline CpG methylation with serum 25(OH)D response		CYP24A1	cg22257236			Body			0.01 ^f^		0.003
								cg18956481			5’UTR, 1stExon			0.01 ^f^		0.005
								cg06368932			1stExon, 5’UTR			0.01 ^f^		0.01
								cg24582168			Body			0.008 ^f^		0.03
								cg17997279			TSS1500			0.01 ^f^		0.04
								cg10574372			Body			-0.006 ^f^		0.04
							CYP27A1	cg15713912			Body			-0.02 ^f^		0.007
								cg13648565			Body			-0.01 ^f^		0.03
								cg13908635			3’UTR			-0.008 ^f^		0.03
								cg14553243			5’UTR, 1stExon			0.009 ^f^		0.04
							CYP2R1	cg25454890			1stExon			-0.006 ^f^		0.03
							CYP27B1	cg07060721			Body			0.01 ^f^		0.02
							VDR	cg13173254			Body, 5’UTR			0.02 ^f^		0.005
								cg08726078			Body			0.01 ^f^		0.007
								cg10037049			5’UTR			0.02 ^f^		0.01
								cg07290465			Body			0.007 ^f^		0.03
								cg23190711			Body, 5’UTR			0.01 ^f^		0.03
								cg01077720			ExonBnd, Body			-0.009 ^f^		0.04
								cg06487630			Body, 5’UTR			-0.01 ^f^		0.04
								cg16998563			TSS1500			-0.004 ^f^		0.048
Suderman et al., (29)	2016	Cohort (MoBa)	Norway	Mothers and their newborns	Maternal 25(OH)D concentrations (at 18 weeks gestation) and cord blood DNA methylation	819	CYP27B1	cg01182309						7.65E-05	0.6	< 0.001
								cg18413900						-0.0001	0.6	0.04
								cg04321714						0.0001	0.6	0.04
								cg04905829						-0.0001	0.6	0.04
		Cohort (ALSPAC)	UK	Mothers and their newborns	Maternal 25(OH)D concentrations (at 28 weeks gestation) and cord blood DNA methylation	597	CYP24A1	cg17997279						-3.55E-05	0.06	0.003
							CYP27B1	cg04321714						-4.49E-05	0.06	0.003
							CYP27A1	cg02930667						-4.16E-05	0.06	0.004
								cg03460682						-2.43E-05	0.06	0.004
								cg22707705						0.0004	0.2	0.02
								cg01797727						-0.0001	0.2	0.02
Beckett et al., (23)	2016	Cross-sectional	Australia	Elderly age (> 65)	Plasma 25(OH)D level (log(x)) with the methylation status of vitamin D-related genes	80	CYP2R1		102,190	11: 14,912,900–14,913,771 ^g^	Promoter			-0.27 ^i^		0.03
							CYP2R1							-0.2 ^h^		0.03
							CYP2R1					0.05				0.04
							CYP27B1		103,271	12: 58,158,855–58,160,000 ^g^	Body			0.03 ^i^		0.8
							CYP27B1							-0.01 ^h^		0.9
							CYP27B1					No statistically significant association				
							CYP24A1		109,362	20: 52,789,252–52,790,986 ^g^	Promoter			-0.33 ^i^		0.04
							CYP24A1							0.02 ^h^		0.8
							CYP24A1					0.06				0.02
							VDR		103,069	12: 48,298,645–48,299,537 ^g^	Promoter			0.25 ^i^		0.03
							VDR							0.26 ^h^		0.01
							VDR					0.12				0.001
Wang et al., (25)	2018	A mixed case-control and prospective cohort	China	Pulmonary tuberculosis patients as the case group and healthy participants as the control group	Cumulative methylation level at each region with 1,25(OH)_2_D levels	240	CYP24A1	CYP24A1_1 ^j^		52,790,591/52,790,815 ^K^			-0.03			0.6
								CYP24A1_2 ^j^		52,790,767/52,791,019 ^K^			0.07			0.3
							CYP27A1	CYP27A1_1 ^j^		219,646,982/219,646,721 ^K^			0.06			0.4
								CYP27A1_2 ^j^		219,646,810/219,646,561 ^K^			0.08			0.2
								CYP27A1_3 ^j^		219,646,624/219,646,403 ^K^			0.13			0.045
								CYP27A1_4 ^j^		219,646,465/219,646,204 ^K^			0.07			0.3
								CYP27A1_5 ^j^		219,646,286/219,646,037 ^K^			-0.02			0.7
							CYP27B1	CYP27B1_1 ^j^		58,160,882/58,160,619 ^K^			0.02			0.8
								CYP27B1_2 ^j^		58,160,053/58,159,785 ^K^			0.12			0.06
								CYP27B1_3 ^j^		58,159,890/58,159,688 ^K^			0.03			0.6
							CYP2R1	CYP2R1_1 ^j^		14,913,830/14,913,634 ^K^			-0.04			0.6
								CYP2R1_2 ^j^		14,913,505/14,913,273 ^K^			-0.05			0.4
								CYP2R1_3 ^j^		14,913,339/14,913,061 ^K^			-0.05			0.5
								CYP2R1_4 ^j^		14,913,116/14,912,845 ^K^			0.07			0.3
							VDR	VDR _1 ^j^		48,299,590/48,299,323 ^K^			0.01			0.9
								VDR _2 ^j^		48,299,412/48,299,179 ^K^			0.1			0.1
								VDR _3 ^j^		48,299,247/48,299,017 ^K^			0.09			0.1
								VDR _4 ^j^		48,299,106/48,298,885 ^K^			0.02			0.7
								VDR _6 ^j^		48,298,733/48,298,464 ^K^			-0.02			0.8
Zhou et al., (26)	2014	Randomized clinical trial	USA/ non-Hispanic white	white postmenopausal women aged ≥ 55 years	The association between DNA methylation status and adjusted vitamin D response variation	145	CYP2R1	The average methylation level of the 14 examined CpG sites					-0.02			0.03
								-4 C					-0.13			0.03
								+ 28 C					-0.13			0.02
								+ 30 C					-0.19			0.002
								+ 33 C					-0.12			0.046
								+ 40 C					-0.17			0.003
								+ 43 C					-0.15			0.01
								+ 69 C					-0.13			0.03
								+ 80 C					-0.14			0.02
						117	CYP24A1	−342 C					-0.11			0.01
								−293 C					-0.13			0.03

TSS200 within 200 basepairs upstream of the transcription start site, TSS1500 within 1500 basepairs upstream of the transcription start site, UTR untranslated region

^a^ The estimated change in methylation (logit(β)) per 10 ng/mL change in serum 25(OH)D level

^b^ CpG sites

^c^ Mean methylation across the assay

^d^ CpG unit (a fragment of DNA containing one or more CpG sites)

^e^ Represent the difference of DNA methylation associated with low serum 25(OH)D response compared with the high response with adjustment of age, sex, and BMI

^f^ Chromosome: Island Location

^g^ Individual predictive value of DNA methylation status for plasma 25(OH)D levels in a model including all assessed methylation sites (adjusted for vitamin D and calcium intake, age, sex, BMI, cigarette smoking history, alcohol intake, and cumulative irradiance)

^h^ Corrected for age, sex, BMI, cigarette smoking history, alcohol intake, and cumulative irradiance

^i^ Fragment

^j^ Start/stop

**Table 2:** Quality assessment of included cohort and cross-sectional studies using the NIH quality assessment tool

Author, year	1	2	3	4	5	6	7	8	9	10	11	12	13	14	Quality rating
Suderman et al., 2016	Yes	Yes	No	Yes	No	Yes	Yes	Yes	Yes	No	Yes	No	NR	Yes	Good
Beckett et al., 2016	Yes	Yes	NR	Yes	No	No	NA	No	Yes	No	Yes	No	NA	Yes	Fair
Harvey et al., 2014	Yes	Yes	NR	Yes	No	Yes	Yes	No	Yes	No	Yes	No	NR	Yes	Fair
Novakovic et al., 2021	Yes	Yes	NR	Yes	No	No	NA	Yes	Yes	No	Yes	No	NA	No	Good
Wjst et al., 2010	Yes	Yes	NR	Yes	No	No	NA	Yes	Yes	No	Yes	No	NA	Yes	Good
O’Brien et al., 2018	Yes	Yes	NR	Yes	No	Yes	NA	Yes	Yes	No	Yes	No	NA	Yes	Good

NIH: National Institute of Health, NA: not applicable, NR: not reported

**Table 3:** Quality assessment of included case-control studies using the NIH quality assessment tool

Author, year	1	2	3	4	5	6	7	8	9	10	11	12	Quality rating
Wang et al., 2018	Yes	Yes	No	Yes	Yes	Yes	NR	Yes	No	Yes	NA	Yes	Fair

NIH: National Institute of Health, NA: not applicable, NR: not reported

**Table 4:** Quality assessment of included randomized clinical trials using the NIH quality assessment tool

Author, year	1	2	3	4	5	6	7	8	9	10	11	12	13	14	Quality rating
Chen et al., 2019	Yes	No	Yes	Yes	No	Yes	NR	NR	Yes	NR	Yes	No	NR	Yes	Fair
Zhou et al., 2014	Yes	NR	NR	NR	NR	NR	Yes	Yes	Yes	NR	Yes	NR	Yes	No	Fair

NIH: National Institute of Health, NR: not reported

A cohort study on 80 elderly patients assessed the association between 25(OH)D status and methylation level of vitamin D metabolic genes, including CYP24A1, CYP27B1, CYP2R1, and VDR (peripheral blood cells were the source for DNA methylation analysis). The average methylation level among examined CpG sites was low. The methylation level of CYP2R1 and CYP24A1 were positively related together (R^2^: 0.17, p-value < 0.001). Bivariate analysis showed a weak negative correlation of 25(OH)D levels with methylation of CYP2R1 (R^2^: 0.05, p-value = 0.04) and CYP24A1 (R^2^: 0.06, p-value = 0.02), and a positive correlation with VDR (R^2^: 0.12, p-value = 0.001). There was no significant association between the 25(OH)D level and the methylation status of CYP27B1. Also, they evaluated the influence of methyl donor availability on the methylation status of mentioned genes. While the relationship of 25(OH)D levels and methylation of the genes in the presence of additional variables—i.e., serum folate, B12, and plasma homocysteine—were not altered, the potential role of serum folate and B12 in methylation regulation of these genes was discovered. Serum B12 level showed a positive correlation with the methylation status of CYP27B1, and serum folate level was related to VDR methylation. The adjusted model with vitamin D and calcium intake, age, sex, body mass index (BMI), cumulative irradiance, alcohol intake, and cigarette smoking history showed a better predictive value for 25(OH)D level (R^2^; 0.54, *p*-value < 0.001) in comparison to modeling without the inclusion of metabolic vitamin D genes methylation status (R^2^: 0.46, *p*-value < 0.001). In the mentioned adjusted model, CYP2R1 gene methylation status was a significant independent negative (β: -0.2, *p*-value = 0.03) predictor of 25(OH)D level, and VDR gene methylation was an independent positive predictive factor (β: 0.26, *p*-value = 0.005). Although there was no significant predictive value for CYP24A1 methylation individually in the described model, a significant predictive value of the interaction of CYP24A1 gene methylation and vitamin D intake was found (*p* interaction = 0.04). This suggests that increased methylation of CYP24A1 is not the direct cause of low serum vitamin D levels but is the response to increased vitamin D availability (increased intake). The significant interaction between CYP2R1 methylation status and the major determinants of serum vitamin D levels, including vitamin D intake (*p* interaction < 0.001), calcium intake (*p* interaction = 0.003), and cumulative irradiance (*p* interaction = 0.009), suggests that CYP2R1 methylation status regulates the effect of those contributing factors on the vitamin D level modulation. VDR gene methylation is positively associated with 25(OH)D levels in the mentioned model. This association remained when interaction with vitamin D intake was considered (p interaction; 0.04) or when corrected in the methyl donor model (R^2^; 0.21, *p*-value = 0.006). These findings suggest a negative feedback loop for maintaining vitamin D homeostasis [23].

One randomized clinical trial was conducted among 64 overweight/obese African Americans. The role of baseline DNA methylation (extracted from whole blood buffy coat) in the serum 25(OH)D level response to vitamin D supplementation was investigated. Due to the expected serum vitamin D level and actual post-test level, participants were categorized into high-response and low-response groups. Expected levels were estimated by the intervention dose, gender, age, body mass index, baseline serum vitamin D level, and seasonal variation. Twenty CpG sites located in the CYP family genes and VDR showed statistically significant associations with serum 25(OH)D response (Table 1). Also, the methylation level of cg07873128 located in the body region of the oxysterol binding protein like 5 (OSBPL5) gene was negatively correlated with response to vitamin D supplementation. They suggested that hypermethylation of the mentioned CpG site could cause cholesterol and calcium regulation impairment, resulting in decreased response to vitamin D supplementation. However, changes in the methylation level of cg07873128 (OSBPL5) were not associated with changes in serum 25(OH)D level (p-value = 0.6). Also, it is mentioned that only methylation changes of cg06368932 (CYP24A1) were positively associated with the changes in 25(OH)D levels [24].

One case-control study among the Chinese population (healthy participants and patients with pulmonary tuberculosis) sequenced 310 CpG sites in the promoter region of 5 candidate genes (CYP24A1, CYP27B1, CYP27A1, CYP2R1, and VDR). CYP27A1_3 was the only region that was significantly associated with 1,25(OH)_2_D level (r = 0.13, *p*-value = 0.045). They evaluated the correlation of methylation levels of these genes and 25(OH)D serum levels by four different models (Model 1: Cumulative methylation level was calculated by adding the frequency of all CpG sites in each region/ Model 2: Cumulative methylation level was calculated by adding the frequency of statistically significant CpG sites in each region/ Model 3: Inclusion of only hypermethylated CpG sites in the cases and exclusion of CpG sites with an inverse relationship between cases and controls/ Model 4: Inclusion of only statistically significant CpG sites after Bonferroni correction). Cumulative methylation levels of CYP24A1, CYP27B1, CYP27A1, or VDR genes were significantly associated with serum 25(OH)D levels in all four models. Although, CYP2R1 was significantly positively associated only in the third model [25].

One clinical trial among non-Hispanic white postmenopausal women evaluated whether methylation statuses of CYP genes were associated with 25(OH)D serum levels in response to vitamin D supplementation. Calcium and vitamin D (1100 IU/day) intervention on 446 subjects for at least 12 months was conducted. Of them, 18 responders (the highest 12-month increase in serum 25(OH)D) and 18 non-responders (the lowest 12-month increase in serum 25(OH)D) were selected. Methylation levels of the promoter regions of both CYP2R1 and CYP24A1 genes at baseline were significantly higher in non-responders compared to responders. However, no significant differences were found in methylation levels of CYP27B1 and CYP27A1 between responders and non-responders at baseline. It was found that only the CYP24A1 gene experienced methylation reduction in both responders and non-responders after a 12-month vitamin D supplementation. It should be added that time by treatment was also significant for only CYP24A1. A validation study on 145 participants was also conducted to confirm these results. The methylation level of eight CpG sites (among the 14 examined sites in 145 subjects) in the CYP2R1 gene was significantly negatively associated with the serum 25(OH)D increment in a 12-month period. The methylation average of 14 evaluated CpG sites of CYP2R1 was also negatively associated with the increment in 25(OH)D level after 12-month supplementation. Baseline DNA methylation of two CpG sites of CYP24A1 (among the 16 examined sites in 117 subjects) was also negatively associated with the vitamin D supplementation response. (Table 1) The contribution of CYP2R1 and CYP24A1 (10 statistically significant CpG sites together) to the vitamin D response variation was reported to be moderate (R^2^ = 6.4%) [26].

In the validation study, methylation reduction in each 14 CpG site of CYP2R1 at the 12-month visit was statistically significant compared to the baseline. It was also mentioned that the decrease in average methylation of the CYP2R1 CpG sites was significant (*p*-value = 0.001). Analysis of the CYP24A1 CpG sites revealed different reactions of the CpG sites to the vitamin D supplementation [26].

DNA extracted from peripheral blood lymphocytes of 384 individuals showed a weak association between CYP27B1 site 15–17 and 25(OH)D levels. Also, there was a tendency for a higher CYP27B1 methylation ratio with a lower 25(OH)D serum level [27].

A sub-cohort study on 1270 non-Hispanic white women examined the association of methylation status of 198 CpGs in or near vitamin D-related genes—i.e., VDR, RXRA (retinoid-x receptor-alpha), GC, CYP24A1, CYP27B1, CYP2R1, and DHCR7/NADSYN1—with vitamin D levels (DNA was extracted from whole blood samples). Among them, 23 CpG sites showed statistically significant associations with 25(OH)D levels. Unexpectedly, it was noted that most of the significant associated CpG sites were located within gene bodies. CpG sites at different parts of the genes showed dissimilar associations with 25(OH)D levels [28].

A 1416 mother/newborn pairs study investigated the association between mid-pregnancy maternal 25(OH)D levels and cord blood DNA methylation. Findings showed a weak association between the methylation status of CpG sites among CYP24A1, CYP27A1, CYP27B1, and CYP2R1 genes and vitamin D levels [29]. (Table 1)

A study on 86 twin pairs and their mothers investigated the relationship between maternal 25(OH)D serum level (at 28-week gestation), placental methylation of CYP24A1, and neonatal cord blood 25(OH)D concentration. Findings showed no correlation between placental CYP24A1 gene methylation level and maternal or neonatal 25(OH)D serum level. Also, it was mentioned that there was no association between CYP24A1 methylation changes and changes in maternal or neonatal 25(OH)D concentration[30].

One study investigated the relationship between maternal 25(OH)D status (measured at 34 weeks gestation) and the methylation status of the RXRA gene retrieved from the umbilical cord. Findings showed that maternal free vitamin D index (the ratio of serum 25(OH)D to vitamin D binding protein concentration) had a statistically significant negative association with RXRA CpG4/5 methylation percentage (β = -3.29 SD/unit, p-value = 0.03). However, the results showed that 25(OH)D or vitamin D binding protein serum level was not a predictive factor for the methylation status of any site at RXRA [31].

## Discussion

Vitamin D affects the epigenome on multiple levels and has a substantial role in the epigenetic regulation of genes [19, 32]. On the other hand, epigenetic mechanisms could regulate vitamin D metabolites [32, 33]. Therefore, it could be suggested that pathologies that demonstrated any association with epigenetic effects of vitamin D metabolites could be attributed to complex processes involving epigenetic modulation of genes engaged in vitamin D metabolism [34]. The present study illustrated that the epigenetic modulation of vitamin D-related genes could be the reason for vitamin D level variance among the population. In addition to its role in normal variation, it can be a reason for vitamin D deficiency. Several studies described the association between the methylation status of those genes—CpG islands located at the promoters and within the gene locus [32]—and vitamin D levels. According to the studies, the methylation status of CYP2R1, CYP27B1, CYP24A1, and VDR genes is responsible for nearly 18% of the vitamin D level variance [23].

CYP2R1 methylation status can regulate the effect of the contributing factors (e.g., calcium and vitamin D intake, cumulative radiance) on vitamin D serum levels [23]. A study on African-American adolescents showed that the CpG site at the CYP2R1 gene showed lower methylation in participants with sufficient levels of vitamin D in comparison with the participants with vitamin D deficiency [35]. Also, a significant reduction in the mean methylation level of CYP2R1 CpG sites following a period of vitamin D supplementation was reported [26]. Most of the studies confirmed that the methylation status of CYP24A1 is regulated by the vitamin D level. Studies suggested that impaired methylation of CYP24A1 occurred in response to increased 25(OH)D serum levels [23, 26]; however, another study suggested that impaired methylation of CYP24A1 was a direct cause of 25(OH)D deficiency [35]. A clinical study showed that the increase in the 1,25(OH)_2_D level could increase the activity of CYP24A1 over a few hours [14]. Another study showed that in response to 1,25(OH)_2_D, a 6-8-fold increase in VDR and RXR at the promoter of CYP24A1 and a 3-fold increase in H4 acetylation of coding and promoter regions were observed in the mice [18]. Therefore, these all together result in the initiation of CYP24A1 transcription in response to increased vitamin D availability.

A weak association between higher methylation of the CYP27B1 gene and lower 25(OH)D serum level is shown [27]. This could mean that in case of 25(OH)D availability, CYP27B1 would be more translated. Contrarily, a study on individuals aged 65 years or more found no direct correlation between plasma 25(OH)D and the methylation status of CYP27B1 [23]. In one case-control study on 122 patients with pulmonary tuberculosis and 118 healthy controls, the methylation of a fragment (cumulative methylation of CpG sites at the specific region of the gene) at the CYP27A1 showed a positive correlation with 1,25-dihydroxyvitamin D level [25] which could suggest the inhibitory role of 1,25(OH)_2_D on 25-hydroxylation.

Vitamin D binding protein (DBP) (encoded by the GC gene) binds to approximately 85% of circulating vitamin D metabolites [8]. In one of the reviewed studies, the methylation status of 3 CpG sites at the GC gene showed statistically significant positive beta coefficients for serum 25(OH)D level [28]. Although variation in DBP level can result in variation of total 25(OH)D level, free 25(OH)D serum level does not change. Therefore, lower DBP cannot cause vitamin D deficiency symptoms even in undetectable amounts of 25(OH)D (supporting the free hormone hypothesis) [8].

Studies showed the correlation between RXRA and VDR genes’ methylation status and serum vitamin D levels. The epigenome-wide association study revealed 8 CpG sites at the RXRA gene that were significantly associated with serum 25(OH)D level [28]. A cross-sectional study described the positive correlation between VDR methylation status and plasma 25(OH)D level independently and when corrected for the effect of vitamin D intake or methyl donor serum bioavailability. Results showed the negative feedback mechanism between 25(OH)D level as a ligand and VDR as a receptor [23].

Despite the fact that some genes do not directly influence the vitamin D metabolic pathway, they can affect the serum level of vitamin D metabolites and therefore lead to vitamin D deficiency symptoms. A study on overweight/obese African Americans revealed a negative correlation between the baseline methylation level of a CpG site located in the body of the OSBPL5 gene and response to vitamin D supplementation [24]. It is considered that hypermethylation of the CpG site located in the body of the gene would downregulate the transcription of the OSBPL5 gene [24, 36]. However, there are inconsistent findings related to the association between methylation of CpG sites located in the body of the genes and transcription [28, 37].

NADSYN1 (nicotinamide adenine dinucleotide synthetase 1) gene, which is located near DHCR7, has no known direct biological effect on vitamin D metabolism [38]. However, a genome-wide association study (GWAS) found a strong association between SNPs in the DHCR7/NADSYN locus and serum level of vitamin D metabolites [39]. One reviewed study revealed 7 CpG sites at the NADSYN1 gene that showed statistically significant associations with 25(OH)D levels. Also, they discovered that methylation levels of 4 CpG sites at the DHCR7 gene showed statistically significant associations with 25(OH)D levels, and three of them showed positive beta coefficients [28]. (Table 1) Another genome-wide methylation study showed two CpG sites at the DHCR7 gene with significantly different methylation levels between vitamin D deficient participants and participants with desirable vitamin D levels. However, CpG sites showed different methylation patterns [35].

The change in 25(OH)D serum level after vitamin D supplementation varies among individuals. Some factors, including body weight, age, sex, type of vitamin D supplementation (D_2_ or D_3_), calcium intake, baseline serum 25(OH)D, and physical activity, might result in this variation. These factors can lead to up to 50% of the changes [40, 41]. A recent review article discussed the use of genetic risk scores (GRS)—which describes combined variants of vitamin D lowering alleles—in assessing the responsiveness to vitamin D supplementation and also recommending the optimal dosage according to the genetic score [42]. Besides genetic variation [43], epigenetics could be another reason for the mentioned differences. One of the reviewed studies suggested that subjects with high methylation rates of the CYP2R1 and CYP24A1 genes may need higher dosages of vitamin D supplementation to achieve optimal serum levels [26]. It was reported that the contribution of the methylation status of CYP24A1 and CYP2R1 could explain 6.4% of vitamin D response variation [26]. They discussed that the absence of an association between CYP27A1 and vitamin D response could be due to its lesser activity in 25-hydroxylation compared to CYP2R1[26]. Another study reported twenty CpG sites located in the VDR and CYP family genes that showed statistically significant association with serum 25(OH)D response [24].

Generally, DNA methylation can be sensitive to cigarette smoking, alcohol usage, BMI, and the availability of methyl donors like folate and B12 [28]. It is reported that the inclusion of serum B12, folate, and plasma homocysteine in the multivariable regression analysis would not alter the correlation of CYP2R1, CYP24A1, and VDR methylation status with plasma 25(OH)D level. However, a potential role for serum B12 and folate in the methylation regulation of CYP27B1 and VDR was reported, respectively [23]. Another study showed that high folate intake could effectively lead to reduced expression of CYP24A1 in the ascending colon [44].

Recently, a new concept called personalized response to vitamin D supplementation has been suggested [45]. The results of clinical trials like VitDbol and VitDmet about the effects of vitamin D supplementation on biochemical vitamin D-sensitive parameters, accessibility change of chromatin regions, and the response of transcriptome-wide vitamin D target genes have been published lately [46–52]. These trials revealed the difference in humans’ molecular response to vitamin D supplementation [45]. According to the fold change of 25(OH)D serum level and fold change of parameters or gene expression, three groups were proposed; low responder, mid responder, and high responder. Individuals as low responders are more susceptible to vitamin D deficiency disorder and should take higher daily doses of vitamin D than high responders to obtain the optimal hormonal activity of vitamin D and maximal disease protective effect [11, 45]. So, personalized vitamin D supplementation based on the personalized optimum vitamin D level can be considered instead of a general recommendation. It is mentioned that this index is not related to the geographic location of individuals and is independent of the serum 25(OH)D levels [45]. Furthermore, a review study on Mendelian Randomization studies suggested that the benefits of increased vitamin D levels could be attributed to the correction of clinical deficiency [42]. Therefore, it can be concluded that the evaluation of vitamin D serum levels based on general threshold metrics could not be a good indicator of the optimal vitamin D level for the desired effects.

It is suggested that genetic variation can only predict 20% of the variation in vitamin D response indices, while the remaining could be due to epigenetic variations [45]. We suggest that this variation can be due to the differences in the functional status of intracellular 25(OH)D transformers or regulation of VDR, RXRA, and the co-regulators. It can be hypothesized that variations in the amount or function of the megalin (which transfers DBP-bound format to cells) and HSP70 (the potential intracellular transporter) could be possible reasons for vitamin D response variation [8].

The use of PBCs as the source of DNA methylation analysis is a major limitation of the reviewed articles. Although the expression of the mentioned enzymes in immune cells is demonstrated, the regulation of vitamin D status majorly occurs in liver and kidney tissues. Furthermore, the presence of CYP27B1 regulation via 1,25(OH)_2_D/VDR in renal cells and the absence of it in macrophages—due to genetic and epigenetic mechanisms that result in tissue-specific actions of 1,25(OH)_2_D—points to this error in the generalizability of the results [8].

According to the discrepancy in the method of studies, different populations, and methods of vitamin D level and methylation status assessment, reviewed studies showed diversity in the correlated CpG sites of each gene and their association with vitamin D level. We suggest large-scale studies in different ethnicities to find the effect of epigenetic modulation of vitamin D-related genes on vitamin D response variation and serum level. It can determine the usability of the epigenetic profile for the recommendation of the appropriate vitamin D supplementation dosage. Afterward, we could analyze each person’s metabolic response to the vitamin D serum level to determine the goal serum level.

## Conclusions

Epigenetic modification plays a crucial role in regulating vitamin D levels and vitamin D response variation. Epigenetic changes could be considered to recommend the appropriate dosage of vitamin D supplementation. Large-scale research studies among various ethnicities are recommended to report more precisely.

## Electronic supplementary material

Below is the link to the electronic supplementary material.

Supplementary Material 1

## Data Availability

Not applicable.

## Abbreviations

NIH: National Institutes of Health
25(OH)D: 25-Hydroxyvitamin D
7-DHC: 7-Dehydrocholesterol
NADSYN1: Nicotinamide adenine dinucleotide synthetase 1
DHCR7: 7 Dehydrocholesterol Reductase
VDR: Vitamin D receptor
1,25(OH)_2_D: 1,25-Dihydroxyvitamin D
RXR: Retinoid X receptor
FDR: False discovery rate
OSBPL5: Oxysterol binding protein like 5
DBP: Vitamin D binding protein
BMI: Body mass index
CYP2R1: Cytochrome P450 Family 2 Subfamily R Member 1
CYP27A1: Cytochrome P450 Family 27 Subfamily A Member 1
CYP3A4: Cytochrome P450 Family 3 Subfamily A member 4
CYP27B1: Cytochrome P450 Family 27 Subfamily B Member 1
CYP24A1: Cytochrome P450 Family 24 Subfamily A Member 1
