# Supplementary material for: Effect of epigenetics on vitamin D levels: a systematic review until December 2020
Source: Arch Public Health. 2023 Jun 15;81:106. doi: 10.1186/s13690-023-01122-2 (PMC10268530; doi:10.1186/s13690-023-01122-2)
Supplement: Supplementary file 1 — Supplementary Material 1 [file 13690_2023_1122_MOESM1_ESM.docx]

**Aims and Scope statement**

1. What is known?

Vitamin D is a fat-soluble secosteroid with crucial roles in calcium hemostasis and different extra-skeletal pathways. The production and metabolic pathways of vitamin D and its activated metabolites consist of several enzymes which are encoded by coding genes. Epigenetics denotes the process of alteration in gene activity without any change in the gene sequence. Epigenetics and vitamin D status is a novel field of research. Although the epigenetic effect of vitamin D on the transcription of target genes has been discussed, the association between epigenetic modification of the genes involved in the vitamin D metabolic pathway and vitamin D metabolites’ status has been elucidated incompletely.

1. What does the study add?

This systematic review aimed to shed light on the effect of epigenetic alteration -methylation level or its changes- of genes involved in vitamin D regulation on the vitamin D metabolites’ serum level or their changes. According to the literature, it is the first systematic review that specifically explored this association.

Studies illustrated that epigenetic modulation of vitamin D-related genes could be the reason for vitamin D level variance among the population. In addition to its role in normal variation, it could be the reason for vitamin D deficiency. According to the studies, the methylation status of CYP2R1, CYP27B1, CYP24A1, and VDR genes is responsible for nearly 18% of the vitamin D level variance.

Reviewed studies reported lower methylation levels of the CpG site at the CYP2R1 gene in individuals with sufficient levels of vitamin D in comparison with the participants with vitamin D deficiency. The regulatory role of CYP2R1 methylation status in response to factors like calcium and vitamin D intake was also reported. Most of the studies confirmed that the methylation status of CYP24A1 is regulated by the vitamin D level. Studies showed the negative feedback mechanism between 25(OH)D level as a ligand and VDR as a receptor.

The change in 25(OH)D serum level after vitamin D supplementation varies among individuals. Besides age, sex, type of vitamin D supplementation (D2 or D3), calcium intake, baseline serum 25(OH)D, and physical activity, epigenetics could be another reason for the mentioned difference. One of the reviewed studies suggested that subjects with high methylation rates of the CYP2R1 and CYP24A1 genes may need higher dosages of vitamin D supplementation to achieve optimal serum levels.

Recently, a new concept called personalized response to vitamin D supplementation has been suggested. Individuals as low responders are more susceptible to vitamin D deficiency disorder and should take higher daily doses of vitamin D than high responders to obtain the optimal hormonal activity of vitamin D and maximal disease protective effect. It is suggested that genetic variation can only predict 20% of the variation in vitamin D response indices, while the remaining could be due to epigenetic variations.

1. What are implications for clinical practice, public health, and/or research?

Authors suggest large-scale studies to explore the effect of epigenetic modulation of vitamin D-related genes on vitamin D response variation and serum level. Furthermore, researchers could investigate the usability of the epigenetic profile for the recommendation of the appropriate vitamin D supplementation dosage. Afterward, we could analyze each person’s metabolic response to the vitamin D serum level to determine the goal serum level that each individual should reach.
